# Two-Way FDI assists agricultural sustainable development: Based on digitalization and greening perspectives

**DOI:** 10.1371/journal.pone.0296896

**Published:** 2024-02-16

**Authors:** Tingwei Chen, Feng Yang, Yujie Li, Zongbin Zhang

**Affiliations:** School of Economics, Shandong Normal University, Jinan, Shandong, China; National Technical University of Athens: Ethniko Metsobio Polytechneio, GREECE

## Abstract

With the new challenges and crises facing agriculture, digitalization and green transformation have become important ways to solve the problems. This paper uses an international economics perspective to chart a new path for sustainable agricultural development. Specifically, it analyzes whether two-way international direct investment (FDI) can facilitate agricultural digital-green fusion(DGF)? Using a sample of 31 provinces (autonomous regions) from 2012 to 2021, this study finds: (1) Two-way FDI can significantly contribute to agriculture’s DGF. (2) In the mechanism test, it is proved that two-way FDI can promote agriculture’s DGF level by promoting green technology innovation capacity and overall regional technology innovation capacity. (3) The positive effects of two-way FDI are prominent in the eastern and central regions, coastal regions, and economically developed areas. (4) In the spatial Durbin model, the local two-way FDI growth improves agriculture’s DGF level in the surrounding areas to a certain extent. The government is advised to prioritize openness, foster an environment for technological innovation, leverage spatial radiation for agricultural DGF, and advance digitally empowered agricultural modernization.

## 1. Introduction

### 1.1 Research background

Under the background of a wide range of new information technology application scenarios, the promotion of new information technology on agricultural development represented by artificial intelligence (AI), big data, cloud computing and blockchain technology has also become increasingly prominent, making society enter the era of digital agriculture [1–4]. In this era, digital technological innovations under data-driven mechanisms have increased social productivity and enabled more precise decision-making. This evolution promotes a more dynamic, precise and responsive management decision-making framework that facilitates the digital transformation of agriculture [5]. The development of the digital economy is driving more precise technological advances, of which green innovation technology advances have become an important foundation [6]. Green innovation is a key force for economic and social excellence by controlling environmental pollution, mitigating environmental damage, utilizing natural resources in an appropriate and rational way, enhancing the competitiveness of primary industries, driving economic momentum and promoting social equity [7]. The adaptability and holistic attributes of digital technologies offer new perspectives on digital transformation and green innovation in agriculture as we promote sustainable development and modernization of agriculture [8, 9]. This study proposes the groundbreaking concept of DGF in agriculture, emphasizing the embedding of digital technologies in green innovation frameworks rather than merely technological advances that stimulate green innovation. It goes beyond isolated perspectives and proposes a modern paradigm that resonates with current shifts [6]. The convergence of digital technology and green innovation marks a critical moment in the evolution of agriculture towards refinement, eco-friendliness and intelligence. This convergence is crucial to accelerating structural system reform, opening up technological routes and improving development blueprints.

### 1.2 Research motivation

In recent years, China has actively fulfilled the Paris Agreement and put forward the ambitious goal of “30.60” [9, 10]. Under the guidance of the five core development concepts of “Innovation, Coordination, Green, Openness and Sharing”, China’s economy is moving towards refined growth [11]. The Chinese government released a White Paper on Green Development in a New Era and a Digital Agricultural and Rural Development Plan (2019–2025), demonstrating China’s unwavering commitment to improving the quality and sustainability of its economic development. From 2012 to 2019, China’s Agricultural Green Development Index rose from 73.46 to 77.14, an increase of 5.01%. During the same period, the level of digitization of China’s agriculture and rural areas has increased, and the new rural digital economy has flourished. In this context, Chinese agriculture must integrate digital technologies into sustainable development strategies and prioritize environmental management while enhancing innovation. The implementation of China’s “bringing in” and “going out” strategies has contributed to a significant increase in China’s import and export trade, as well as growth in China’s outward foreign direct investment (IFDI) and non-financial outward foreign direct investment (OFDI). This has provided impetus for the movement of factors of production such as labor, land, capital, technology and information, facilitating the domestic and international circulation of the Chinese economy. As a result, it favors the growth of the Chinese economy and the world economy. An important way to achieve sustainable agricultural development is to improve DGF of agriculture. With the continuous development of two-way FDI, it has become crucial to analyze whether two-way FDI can promote the agriculture’s DGF level and to understand the factors influencing it. Therefore, from both theoretical and practical perspectives, this paper aims to explore whether two-way FDI can promote the level of DGF in Chinese agriculture, its mechanism and spatial effects. The answers to these questions have important theoretical and practical implications for developing countries to formulate more effective international investment policies to promote agricultural digital transformation and green economic growth.

## 2. Literature review

Digitization and greening play a pivotal role in achieving sustainable economic development. With the rise of the digital economy, there has been extensive academic exploration, particularly in the area of digitization and sustainable development. However, many of these studies have been isolated. Conceptually, digital technology is the convergence of information, computing, communication and connectivity pathways, encompassing platforms and tools [12]. Digital transformation depends on the deployment of digital technologies, which can translate into product innovation, enhanced production methods, and the metamorphosis of organizational and business models [8, 13]. Green innovation promotes sustainable approaches that harmonize economic and social needs with environmental management. While existing studies have clearly delineated each, they have yet to define the emerging impacts of their interactions. A key research gap is how to accurately describe the conceptual content of the convergence of digitalization and greening in agriculture and the factors that influence it.

### 2.1 Digitization of agriculture

Existing literature on digitization in agriculture covers a number of aspects, including the concept, current status of development, path of action and relevance. Digitization refers to the use of digital technologies to transform business models and create new opportunities for income generation and value addition. The core of digitization is the application of technology to convert analog information into digital form. Furthermore, digitization is recognized to have a profound impact on companies, upstream and downstream businesses, networks, and ecosystems [14–16]. Onitsuka et al. (2018) [17] observed that the integration of the digital economy and rural industries, following the Japanese government’s introduction of the Sixth Industrial Development Goal for Agriculture, Mountains, and Fishing Villages in 2008, can lead to a multiplier effect, enhancing the international competitiveness of agricultural products, promoting the development of rural economies, and increasing farmers’ income. Zhang Hong et al. (2021) [18] constructed an evaluation index system for the high-quality development of digital agriculture in China, focusing on six aspects. The study revealed a gradual improvement in the level of digital agriculture development, along with significant variations among provinces, characterized by a spatial distribution pattern of ‘East-Middle-West’, indicating varying levels of development from high to low. Human resources play a critical role in the innovative path of agricultural digital transformation. Changes in human resource elements within the agricultural sector can enhance the level of human capital and facilitate the effective utilization of digital technologies in rural areas for production activities [19]. Moreover, the successful implementation of digital agriculture requires a substantial number of highly skilled individuals such as experts, technicians, and farmer service providers. Additionally, it necessitates the development of production and sales capabilities at a certain scale to cater to the requirements of the agricultural and food industries [20–22]. Xia Xianli et al. (2019) [23] assert that the prospective goal of high-quality agricultural development must be market-oriented. The digital transformation of agriculture is driven by market demand, and the construction of ‘digital villages’ is built upon digital technology.

### 2.2 Greening of agriculture

Most of the existing analyses focus on green total factor productivity, and there are limited studies specifically focusing on the greening of agriculture. Therefore, this paper conducts a literature review and analysis in the direction of agricultural greening. It includes macro-level studies on the connotation, measurement criteria and influence mechanisms of green development, as well as micro-level studies related to green agricultural production at the individual level. Green development in agriculture entails the application and expansion of the green development concept in the agricultural sector, continuing and deepening the goal of sustainable agricultural development in the new era, and affirming and integrating ecological agriculture and green agricultural models [24]. Scholars have made significant contributions to the construction of an evaluation index system for green development in agriculture and the analysis of evaluation results. For instance, Zhao Huijie et al. (2019) [25] utilized the triple benefits of economy, society, and ecology as primary indicators and derived specific quantitative hierarchical indicators such as resource conservation, environmental friendliness, ecological conservation, and quality efficiency. The study reveals that the level of green development in Chinese agriculture is gradually increasing, although variations among provinces and regions are more apparent. Yu Fazhan (2018) [26] argued that addressing the low utilization efficiency and pollution associated with the two fundamental and core ecological resource elements of agricultural production—arable land and water—is key to promoting green agricultural development. Sun Xiaoyan et al. (2019) [27] suggested that arable land intensification, represented by land trust, can foster green agricultural production. In terms of agricultural green production at the micro-farmer level, scholars have provided insightful research findings encompassing basic connotations, behavioral motivations, and path choices. Firstly, Li Mingyue et al. (2020) [28] proposed that agricultural green production refers to a novel production method that achieves resource conservation, pollution reduction, output efficiency, and sustainable development through scientific farming techniques and meticulous field management. Additionally, Li Qingjiang et al. (2014) [29] argued that the goal is to reduce consumption, energy usage, and emissions, thereby achieving the harmonization of economic, ecological, and social "three-dimensional" benefits. Secondly, Dong Ying et al. (2019) [30] contended that the extent to which farmers’ production behavior can be "greened" determines the accomplishment of the objective of green agricultural development. Genius et al. (2014) [31] asserted that it should also encompass information exchange, neighborhood effects, clan kinship, and other factors at the social network level, as well as land trust and cooperative drive at the organizational level. By delving deeper into these behavioral motivations, effective approaches can be explored to regulate farmers’ green production behavior and achieve green agricultural development. Lastly, the path of agricultural green production based on farmers’ behavioral motivations should encompass the establishment of an agricultural green production policy system, an agricultural green production organizational system, the creation of a robust social network for agricultural green production, and the stimulation of the internal drive for farmers’ participation in agricultural green production [32]. The main points are summarized through the above analysis and are detailed in Table 1.

**Table 1:** Summary of views from literature review.

Study	Key Findings
Onitsuka et al.(2018) [17]	The integration of the digital economy and rural industries can bring about a multiplier effect, enhance the international competitiveness of agricultural products, promote the development of the rural economy and increase farmers’ incomes.
Zhang Hong et al.(2021) [18]	The level of development of digital agriculture has gradually increased, with significant differences between provinces, showing a spatial distribution pattern of "East-Middle-West", displaying different levels of development from high to low.
Sadjadi EN et al.(2023) [19]	Changes in human resource factors in the agricultural sector can enhance the level of human capital and promote the effective use of digital technologies for productive activities in rural areas.
Xia Xianli et al.(2019) [23]	The digital transformation of agriculture is driven by market demand, and the construction of "digital villages" is based on digital technology.
Zhao Huijie et al.(2019) [25]	The level of green development in Chinese agriculture is gradually increasing, but the differences between provinces and regions are more obvious.
Yu Fazhan (2018) [26]	Inefficient use of arable land and water and pollution are key to advancing the greening of agriculture.
Sun Xiaoyan et al.(2019) [27]	The intensification of arable land, represented by land trust, can promote green agricultural production.
Li Mingyue et al.(2020) [28]	Green agricultural production refers to a new type of production method that realizes resource conservation, pollution reduction, high output efficiency and sustainable development through scientific farming techniques and refined field management.
Li Qingjiang et al.(2014) [29]	The goal of green production in agriculture is to reduce consumption, energy consumption and emissions, thereby realizing the harmonization of economic, ecological and social "three-dimensional" benefits.
Dong Ying et al.(2019) [30]	The degree of "greening" of farmers’ production behavior determines the extent to which the goal of greening agriculture is accomplished.
Genius et al.(2014) [31]	Through in-depth research on behavioral motives, effective ways to regulate farmers’ green production behaviors and achieve green agricultural development can be explored.
Li CX et al.(2022) [32]	The path of agricultural green production based on farmers’ behavioral motivation should include the establishment of a policy system for agricultural green production, an organizational system for agricultural green production, the creation of a sound social network for agricultural green production, and the stimulation of farmers’ endogenous motivation to participate in agricultural green production.

### 2.3 Research contribution

Despite the growing relevance of digitization and green initiatives, the intersection between them remains to be explored, particularly with regard to mechanisms for converging the two areas. As technology evolves, it becomes increasingly necessary to merge the two areas [6, 33]. Therefore, this study takes 31 provinces (autonomous regions) in China as samples from 2012 to 2021. By constructing a two-way fixed-effects model and a spatial Durbin model, this study delves into the conceptual features, mechanisms, and emerging trends of agricultural DGF. The main contributions of this paper are as follows:(1) From a research perspective, we examine how to achieve simultaneous digitalization and greening of agriculture from an international economics perspective. This study focuses on sustainable development of agriculture, leading to pragmatic and reliable solutions. This unique approach makes a pioneering contribution at the intersection of international economics and agricultural economics. It is probably the first survey to explore the relationship between international direct investment, agricultural DGF. It not only advances the interdisciplinary dialogue, but also emphasizes the critical importance of this work in the broader context of international economics. (2) On the theoretical front, this study not only strengthens the existing understanding of how two-way FDI can contribute to sustainable agricultural development, but also provides new insights into the integration of agricultural development into the emerging “double-cycle” development paradigm. In addition, by examining the transmission mechanisms from the perspective of technological innovation, this study opens new avenues for the development of policies aimed at fostering the level of agricultural DGF. (3) In terms of findings, this study empirically demonstrates not only the positive contribution of two-way FDI to the level of DGF, but also reveals the existence of positive spillover effects. In addition, the analysis of this study emphasizes that two-way FDI improves DGF level by catalyzing the innovation capacity of green technology and the overall technological innovation capacity in the region.

The paper is organized as follows: section 2 outlines the Literature Review; section 3 is Methodology and Analysis; section 4 details the Results; section 5 presents the Discussion; section 6 discusses Conclusions and Future Research Orientations.

## 3. Methodology and analysis

### 3.1 Two-way FDI and agricultural DGF

In the 1980s, Dunning’s foreign investment development path theory proposed that the interaction effect of IFDI and OFDI would be significantly enhanced as the level of economic development increased. According to this theory, IFDI serves as the foundation for OFDI, while OFDI provides strong support for the higher development of IFDI [34]. When considering IFDI and OFDI separately, the host country often attracts IFDI inflows based on market size, which may lead to a reliance on low-end technology and restrict the acquisition of core technologies needed for digital economy development. Similarly, if the host country attracts IFDI by utilizing the environment as a cheap factor input, it may face difficulties in improving environmental conditions, potentially exacerbating energy consumption and pollutant emissions as the demand for energy from IFDI increases. However, the coordinated development of IFDI and OFDI can bring about complementary effects that contribute to the improvement of agricultural DGF. When a country simultaneously receives IFDI and engages in OFDI activities, the capital brought by foreign investment compensates for the host country’s capital accumulation deficiencies and stimulates economic growth. Moreover, as host country enterprises become more developed, they possess better abilities in information collection, screening, and risk identification, which helps mitigate the negative effects of OFDI. Enterprises start investing across borders, establishing subsidiaries, and supporting their parent companies financially. This expansion of production scale enhances their innovation capabilities, reduces energy consumption, and gradually transforms agriculture into a digital and green sector, thereby promoting the digitalization and greening of agriculture. In summary, the coordinated development of IFDI and OFDI, along with the host country’s level of economic development, plays a crucial role in fostering the digitalization and greening of agriculture. The interaction between these two types of investment can lead to positive outcomes by overcoming the limitations and challenges associated with each approach.

**H1:** Two-way FDI will promote agricultural DGF.

### 3.2 Two-way FDI, technological innovation and agricultural DGF

Innovation is an important influencing factor and sign of agricultural DGF. By increasing the level of opening up to the outside world, the home country will attract a large number of high-quality foreign enterprises to invest, and the technology level and innovation ability of local export enterprises will be greatly improved through the learning and imitation of high-quality foreign enterprises with rich experience in the international market. Meanwhile, local enterprises making IFDI can play the reverse technology spillover effect prompting the home country enterprises to learn through "learning by doing" and learning advanced foreign technologies, thus promoting agricultural DGF. Therefore, improved innovation capacity contributes to the digitization of agriculture and also to the development of green agriculture, and moreover to the coordinated development of agricultural DGF. Specifically, the high technology carried by IFDI inflow can significantly drive up the technological progress and management level of the host industry through demonstration effect. In addition, multinational enterprises attach more importance to staff training. The inter-industry linkage brought by the entry of foreign-invested enterprises will strengthen the backward and forward linkage with upstream and downstream enterprises, and through providing training to the employees of upstream and downstream enterprises, the inter-industry spillover of knowledge will be generated by using the intermediate goods input linkage, thus improving agricultural DGF. Secondly, OFDI has greater autonomy compared with IFDI, so OFDI will exert reverse technology spillover effects through mechanisms such as R&D cost sharing, peripheral R&D divestiture, reverse technology transfer and technology results feedback. Moreover, enterprises carrying out OFDI will inevitably face the competitive pressure from foreign enterprises, which stimulates their willingness to secure their competitive advantages in foreign markets and adopt a technology-biased development mode, and then make technology feedback to the home country through the reverse channel, thus promoting technological innovation in the home country. Finally, the coordinated development of two-way FDI has complementary technology spillover effects. Although IFDI may inhibit the level of green development to some extent, the economic prosperity brought by IFDI is conducive to OFDI development in the host country and will increase the reverse technology spillover effect of OFDI. IFDI is a prerequisite for OFDI development, and in turn, OFDI is a driving force for higher quality development of IFDI [35]. Due to the transfer of excess capacity in the OFDI process, the economic strength of the home country is enhanced, which raises the access threshold of the home country to IFDI and facilitates the flow of highly skilled personnel and the technology spillover effect, thus promoting agricultural DGF Accordingly, this paper proposes the following hypothesis:

**H2:** Two-way FDI can promote agricultural DGF through technological innovation.

**Fig 1:**
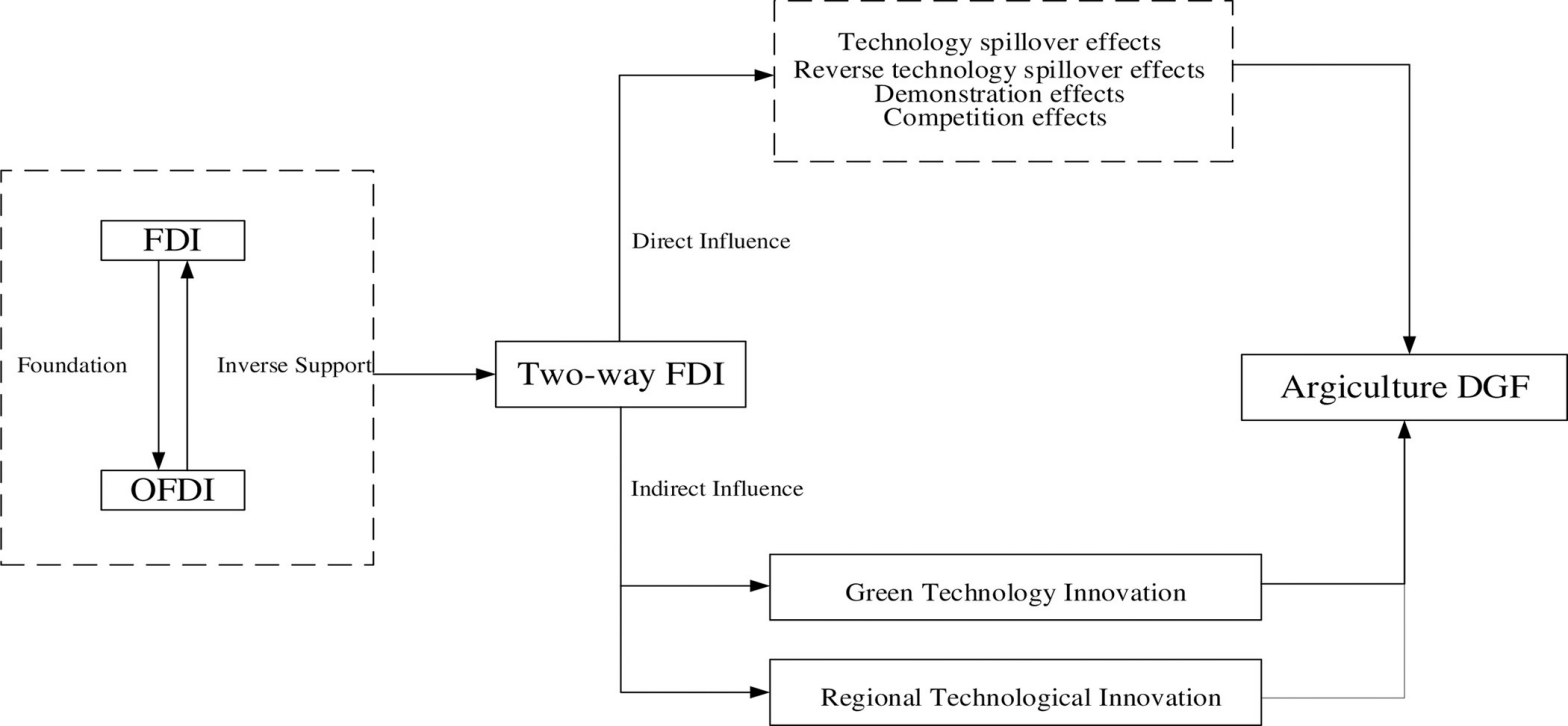
Mechanism analysis framework.

Following the above discussion, this study constructs a mechanism analysis framework to investigate in depth the direct and indirect impacts between two-way FDI and agricultural DGF (Fig 1).

### 3.3 Models and data

#### 3.3.1 Empirical model

1. Panel fixed-effects model
  In order to comprehensively study the impact of two-way FDI development agricultural DGF, this paper draws on the research idea of Zhou Guofu et al. (2023) [36] to construct the following benchmark regression model.
$$DGF_{it}=\alpha_{0}+\alpha_{1}CDFDI_{it}+\alpha_{2}X_{it}+\mu_{i}+\varphi_{t}+\varepsilon_{it}$$ (1)
$$DA_{it}=\beta_{0}+\beta_{1}CDFDI_{it}+\beta_{2}X_{it}+\mu_{i}+\varphi_{t}+\varepsilon_{it}$$ (2)
$$GA_{it}=\gamma_{0}+\gamma_{1}CDFDI_{it}+\gamma_{2}X_{it}+\mu_{i}+\varphi_{t}+\varepsilon_{it}$$ (3)
  In this regression model, i represents the province and t represents the year. DGF, DA, and GA are the explanatory variables, representing the agricultural DGF, the level of development of agricultural digitalization, and the level of development of agricultural greening, respectively. CDFDI is the core explanatory variable representing the level of two-way FDI. X is a series of control variables, containing the level of urbanization, the level of agricultural mechanization, the level of transportation status, rural electricity consumption, and openness to the outside world. The province fixed utility and year fixed utility are denoted by *μ*_*i*_ and *φ*_*t*_ respectively. *ε*_*it*_ denotes the random error term. *α*, *β* and *γ* measure two-way FDI on the agricultural DGF, on the level of agricultural digitization, and on the level of agricultural greening, respectively. The magnitude and sign of these three coefficients are the focus of this paper.
2. Intermediary Mechanism Model
  To further investigate whether innovation capacity is a potential influence mechanism, the following model is set up to study how two-way FDI affects agricultural DGF:
$$INNG_{it}=\eta_{0}+\eta_{1}CDFDI_{it}+\eta_{2}X_{it}+\mu_{i}+\varphi_{t}+\varepsilon_{it}$$ (4)
$$INNT_{it}=\lambda_{0}+\lambda_{1}CDFDI_{it}+\lambda_{2}X_{it}+\mu_{i}+\varphi_{t}+\varepsilon_{it}$$ (5)

Among them, INNG and INNT are potential mediating variables, which indicate green technology innovation capacity and regional technology innovation capacity, respectively. If the *η* coefficients are significantly positive, it indicates that two-way FDI will promote agricultural DGF through promoting green technology innovation capacity. If the *λ* coefficient is significantly positive, it indicates that the two-way FDI will promote agricultural DGF by promoting the regional technological innovation capacity. The meanings of the remaining variables are consistent with Eq (1).

#### 3.3.2 Variable selection

*3*.*3*.*2*.*1 Dependent variable*. The explanatory variable is agricultural DGF (DGF), and this paper draws on the recent study of Wang Heng et al. (2023) [37] on digitalization and greening of agriculture, and on the basis of comprehensive data availability, the indicator system is modified, and the entropy weighting Topsis method is used to assign weights to digitalization and greening of agriculture respectively, and the corresponding comprehensive The corresponding comprehensive index scores were calculated. Using the coupled coordination model, the agricultural DGF was calculated. The specific index system is shown in S1 File.

*3*.*3*.*2*.*2 Independent variable*. The coupled coordination model in physics is used to measure the level of two-way FDI, and this type of method is characterized by avoiding the problem of multiple covariance that may be caused by the interaction term, while measuring from two dimensions, coupling degree and coordination degree, can better reflect the degree of synergy and cooperation between two-way FDI. Based on the research theme of this paper, the two-way FDI development level at the provincial level is measured by drawing on the formula of Zou and Chen Xun (2021) [38] for measuring the level of coupled and coordinated development of two-way FDI.

$$CDFDI_{it}={\left\{IFDI_{it}*OFDI_{it}/[\frac{IFDI_{it}+OFDI_{it}}{2}]\right\}}^{1/2}$$ (6)

where *IFDI*_*it*_ and *OFDI*_*it*_ denote the amount of foreign direct investment flows and outward direct investment flows in province i in year t. The data are converted to RMB using the annual average price of RMB exchange rate and deflated using the GDP deflator. In addition, the interaction term of IFDI and OFDI flow values after taking logarithms is introduced in this paper and included in the robustness test. Since the values of two-way FDI development levels are taken to be large, this paper uses a reduction of 10,000 times to be included in the equation. The same is true for the interaction term of the logged IFDI and OFDI flow values.

*3*.*3*.*2*.*3 Influence mechanism variable*. In this paper, the number of green invention patents (INNG) is used to represent green technology innovation capability. The data are obtained from the China Research Data Service Platform (CNRDS). Among them, the database fully follows the green patent classification standard of the World Intellectual Property Office for patents originating from the State Intellectual Property Office and Google Patent. The number of granted invention patents is used to represent the overall technological innovation capability (INNT). In order to avoid the interference caused by high skewness, the number of patents is processed by taking the natural logarithm after adding one to the number of patents.

*3*.*3*.*2*.*4 Control variables*. In this paper, several factors that may affect the agricultural DGF are selected as control variables. (1) Level of urbanization (URB): expressed as the ratio of urban population to total population. (2) Level of agricultural mechanization (LAG): expressed as the total power of agricultural machinery, and logarithmically treated. (3) Traffic condition (LOAD): expressed as the sum of railroad mileage and road mileage, and logarithmically processed. (4) Rural electricity consumption (ELE): expressed in terms of agricultural power generation, and logarithmically processed. (5) Openness to the outside world (OPEN): expressed as the ratio of total import and export trade to GNP.

#### 3.3.3 Data sources and descriptive statistics

The relevant data used in this paper are from the China Statistical Yearbook, the China Foreign Investment Bulletin and the statistical yearbooks and statistical bulletins of provinces, cities and autonomous regions, etc., except for the data on green patents which are from the China Research Data Service Platform (CNRDS), the rest of the data are from the China Statistical Yearbook, the China Foreign Investment Bulletin and the statistical yearbooks and statistical bulletins of provinces, cities and autonomous regions in previous years, etc. 31 provinces, cities and autonomous regions in China from 2012 to 2021 are selected as the research objects, and due to the serious lack of data, the Hong Kong, Macao and Taiwan are excluded due to serious missing data. In addition, individual missing data are supplemented by trend extrapolation method. The data in this study are all based on the year 2000 and are deflated by the relevant price indices to facilitate the exclusion of price changes and price factors, except for the ratio data. Table 2 shows the results of descriptive statistics of each variable. From Table 2, the mean value of DGF is 0.566, and the standard deviation is 0.0759. this initially indicates that the degree of integration of agricultural digitization and greening in China is at an intermediate level, and the differences between provinces are small. the mean value of DA is 0.218, and the standard deviation is 0.119; the mean value of GA is 0.522, and the standard deviation is 0.0343. this indicates that the degree of difference in agricultural digitization level of each region is are larger and the degree of greening level is higher. The raw data and codes used in this study are in S2 and S3 Files.

**Table 2:** Variable data source and descriptive statistics.

Variable	N	Mean	SD	Min	Median	Max
DGF	310	0.566	0.0759	0.382	0.565	0.795
DA	310	0.218	0.119	0.0398	0.195	0.752
GA	310	0.522	0.0343	0.435	0.523	0.616
CDFDI	310	0.111	0.0764	0.00280	0.0977	0.354
IOFDI	310	0.0183	0.00450	0.00240	0.0189	0.0267
URB	310	0.585	0.130	0.228	0.571	0.932
LAG	310	-0.456	0.223	-1.035	-0.411	-0.0297
LOAD	310	11.72	0.831	9.473	11.99	12.91
ELE	310	4.769	1.490	0.0169	4.722	7.606
OPEN	310	0.241	0.251	0.00710	0.138	1.358
STRR	310	0.283	0.0224	0.231	0.283	0.356
STRO	310	1.287	0.700	0.549	1.135	5.297
INNG	310	6.069	1.532	0.693	6.219	9.114
INNT	310	8.211	1.581	3.526	8.332	11.54

## 4. Result

### 4.1 Baseline regression analysis

Table 3 reports the estimation results of the impact of two-way FDI on agricultural DGF. Among them, Columns (1) to (3) show the estimated results of the impact of two-way FDI on agricultural DGF and its decomposition when no control variables are included. It can be found that two-way FDI can promote agricultural DGF, and it also has a positive effect on the level of digital agriculture. However, the positive contribution of two-way FDI to the greening of agriculture is not yet significant within the sample study period. Columns (4) to (6) show the estimated results after adding the control variables, and it can be found that for every 1 unit increase in the two-way FDI, agricultural DGF increases by 0.1604 units, and the level of digital agriculture increases by 0.435 units, and the coefficients all pass the 1% significance level test. However, the positive promotion effect of two-way FDI on agricultural greening is not significant. This indicates that two-way FDI promotes the agricultural DGF level and agricultural digitalization level during the sample period. On the one hand, two-way FDI promotes the technological progress of agricultural production through technology spillover effect and reverse technology spillover effect, derives new management modes such as Internet of Things(IoT) [39], relies on convenient IoT system, effectively connects agricultural production and marketing, improves comprehensive agricultural efficiency, and promotes the development of agricultural digitization. On the other hand, two-way FDI achieves efficient utilization of agricultural production resources and promotes the digitalization and green transformation of agricultural production processes by optimizing the efficiency of resource allocation between regions. Therefore, hypothesis 1 is proved.

**Table 3:** Impact of two-way FDI on the DGF and its decomposition.

Variables	(1)	(2)	(3)	(4)	(5)	(6)
	DGF	DA	GA	DGF	DA	GA
CDFDI	0.1640***	0.5435***	0.0501	0.1604***	0.4350***	0.0278
	(4.1686)	(5.3096)	(0.9343)	(4.3043)	(5.5188)	(0.4829)
Constant	0.5475***	0.1577***	0.5161***	0.8533***	1.3239***	1.1547***
	(122.5842)	(13.7831)	(86.5802)	(4.4207)	(3.4917)	(5.6325)
Control Var	no	no	no	Yes	Yes	Yes
Province FE	Yes	Yes	Yes	Yes	Yes	Yes
Year FE	Yes	Yes	Yes	Yes	Yes	Yes
Observations	310	310	310	310	310	310
R^2^	0.9751	0.9542	0.8269	0.9769	0.9600	0.8356

* Values of t-statistics in parentheses are at heteroskedasticity robust standard errors

*, **, and *** are significant at the 10%, 5%, and 1% levels, respectively. Same for the following tables.

### 4.2 Robustness tests

To verify the robustness of the baseline regression results, this paper uses replacing the cluster-to-province level standard errors and replacing the core explanatory variables for robustness analysis. Column (1) of Table 4 shows that the regression coefficient of two-way FDI development level is still significantly positive at the 1% level after replacing the standard errors at the cluster to province level. Column (2) shows that the regression coefficient of two-way FDI development level is still significantly positive at the 1% level after replacing the core explanatory variable measure. Therefore, the estimation results of the baseline regression are proved to be robust.

**Table 4:** Regression results of robustness test.

Variables	(1) Robust standard error for clustering to provinces	(2) Substitution of core explanatory variables	(3) Endogeneity Analysis-2SLS	
	DGF	DGF	CDFDI	DGF
CDFDI	0.1604***			0.3565***
	(3.4286)			(3.9051)
IOFDI		1.5236**		
		(2.1128)		
L. CDFDI			0.3976***	
			(3.8761)	
L2. CDFDI			0.0604	
			(0.6605)	
Constant	0.8533**	0.9218***		
	(2.3726)	(4.3174)		
Control Var	Yes	Yes	Yes	Yes
Province FE	Yes	Yes	Yes	Yes
Year FE	Yes	Yes	Yes	Yes
Observations	310	310	248	248
R^2^	0.9769	0.9757		0.8719

In addition, since the level of two-way FDI development and agricultural DGF are both outcome variables. Therefore, there may be a mutual causality endogeneity problem between the two, so this paper uses the two-stage least squares with lags 1 and 2 of the two-way FDI development level as the instrumental variables, and the estimation results are shown in column (3) of Table 4. The results show that the regression coefficient of two-way FDI development level is significantly positive at the 1% significance level. This indicates that the main findings remain robust after accounting for the endogeneity issue.

### 4.3 Heterogeneity test

Considering the differences in geographical location and land and sea location and economic development level, this paper conducts heterogeneity analysis by means of group regression and interaction term based on eastern, central and western regions, coastal and inland regions, high economic development level and less economically developed regions. In order to comprehensively examine the effect of two-way FDI development level on agricultural DGF.

Columns (1) through (3) of Table 5 report the estimation results of geographic location heterogeneity under grouped regression. It can be found that two-way FDI has a significant contribution to agricultural DGF in the Eastern and Central regions, and a negative but insignificant effect on agricultural DGF in the Western region. In column (4), the coefficient of the interaction term Seadum* CDFDI is significantly positive, indicating that two-way FDI is more able to promote agricultural DGF in coastal provinces. Possible reasons for this are the higher level of green economy development in the eastern seaboard. Against the backdrop of urbanization and income growth, people demand higher environmental quality and influence government environmental regulations and corporate production behaviour through consumption preferences and public opinion pressure; at the same time, it is easier to introduce green production technologies in coastal areas, which significantly contribute to green growth in agriculture through the industrial demonstration effect. In column (5), the estimated coefficient of the interaction term Gdpdum*CDFDI is significantly positive at the 1% statistical level, indicating that two-way FDI is relatively stronger in promoting agricultural DGF in provinces with high levels of economic development compared to provinces with less developed economies.

**Table 5:** Regression results of heterogeneity analyses.

Variables	(1)	(2)	(3)	(4)	(5)
	Eastern Region	Middle Region	Western Region	DGF	DGF
CDFDI	0.1348**	0.2005***	-0.0128		
	(2.5707)	(4.7674)	(-0.0897)		
Seadum* CDFDI				0.1871***	
				(3.9382)	
Gdpdum* CDFDI					0.1835***
					(4.1753)
Constant	0.7874***	0.4395	0.7324**	0.8413***	0.8013***
	(3.4385)	(1.5847)	(2.0063)	(4.3182)	(3.9245)
Control Var	Yes	Yes	Yes	Yes	Yes
Province FE	Yes	Yes	Yes	Yes	Yes
Year FE	Yes	Yes	Yes	Yes	Yes
Observations	110	80	100	310	310
R^2^	0.9806	0.9781	0.9735	0.9770	0.9770

### 4.4 Intermediary mechanism test

This paper draws on the analytical ideas of Jiang Ting (2022) [40] for the analysis of the intermediary mechanism, and the results are shown in Table 6. The analysis result in column (1) is the relationship between two-way FDI and green technology innovation capability. It can be seen that two-way FDI can significantly promote the green technology innovation capability with a coefficient of 2.1358 and is significant at the 1% level. The possible reason is that the digital transformation of agriculture has a knowledge spillover effect on agricultural operators, which improves the production quality of green agricultural products and suppresses the generation of agricultural carbon emissions through the dissemination of green production technologies [41], and at the same time, improves the comprehensive quality of agricultural production operators and helps them to cultivate eco-environmental protection awareness [42], which in turn promotes the green growth of agriculture. Jin Yu et al. (2022) [43] argued that companies with greater green innovation capabilities are more motivated to carry out digital transformation activities, and the rise of digital technology has brought new opportunities for green transformation in manufacturing companies [44]. Thus, it is demonstrated that green technology innovation can lead to the coordinated development of digitalization and greening. Taken together, two-way FDI can achieve regional agriculture’s DGF by promoting the green technology innovation capacity. Column (2) analyzes the influence of two-way FDI development level on technological innovation capability from the regional technological innovation capability. It can be found that the coefficient of two-way FDI is 2.5189 and is significant at the 1% level. Therefore, the level of two-way FDI development can significantly contribute to the improvement of the overall regional technological innovation capability. In summary, hypothesis 2 holds.

**Table 6:** Regression results for the intermediary mechanism.

Variables	(1)	(2)
	INNG	INNT
CDFDI	2.1358***	2.5189***
	(4.8960)	(5.9449)
Constant	-6.5112*	3.3979
	(-1.7335)	(1.0667)
Control Var	Yes	Yes
Province FE	Yes	Yes
Year FE	Yes	Yes
Observations	310	310
R^2^	0.9874	0.9902

## 5. Discussion

Emerging information technology innovations such as Artificial Intelligence (AI), Big Data, Cloud Computing, and Blockchain technologies advocate a more dynamic, precise, and responsive management decision-making framework [5], facilitating the digital transformation of agriculture. The adaptive and holistic attributes of digital technologies offer fresh perspectives on digital transformation and green innovation in agriculture as it promotes sustainable development and modernization and transformation [8, 9]. In recent years, China has actively fulfilled the Paris Agreement and put forward the ambitious goal of "30.60" [9, 10]. The Chinese government has issued documents such as the Strategic Plan for Rural Revitalization (2018–2022), the Plan for Digital Agriculture and Rural Development (2019–2025), and the National Plan for Green Agricultural Development in the 14th Five-Year Plan. These documents put forward policies such as "implementing the digital countryside strategy", "carrying out evaluation of green agricultural development", "accelerating the standardization of digital countryside", and "studying and formulating a development evaluation index system". From 2012 to 2019, China’s agricultural green development index rose from 73.46 to 77.14, an increase of 5.01%. Similarly, during the same period, the level of digitization of China’s agriculture and rural areas increased, and the new rural digital economy flourished. The implementation of China’s "bringing in" and "going out" strategies has contributed to the significant growth of China’s import and export trade, as well as the growth of China’s IFDI and OFDI. This has provided impetus for the flow of factors of production, such as labor, land, capital, technology and information, and facilitated the domestic and international circulation of China’s economy. An important way to achieve sustainable agricultural development is to improve the synergy between digitalization and greening of agriculture. With the continuous development of two-way FDI, it has become crucial to analyze whether two-way FDI can promote the level of synergy between digitization and agricultural greening and to understand the factors influencing it. Therefore, this paper aims to explore the level of synergy, the mechanism of action, and the spatial effects of whether two-way FDI can promote the digitalization and greening of agriculture in China from both theoretical and practical perspectives. The answers to these questions have important theoretical and practical implications for developing countries to formulate more effective international investment policies to promote agricultural digital transformation and green economic growth.

This paper finds some interesting conclusions by examining the level of integration of agricultural digitization and greening. Two-way FDI can enhance the level of integration of agricultural digitization and greening, as well as the level of agricultural digitization. But, the effect on the improvement of agricultural greening level is not yet obvious. Through analysis, we believe that the possible reason is that the green growth of agriculture is driven by the transformation of agricultural digitization, and the driving effect of the current development of agricultural digitization on the green development of agriculture in China has not yet reached a critical point. The current digital transformation of Chinese agriculture is characterized by three main features. First, the improvement in the level of rural infrastructure has brought about an increase in productivity and a reduction in production costs, as well as a reduction in energy consumption and environmental pollution [45, 46]. Secondly, the integration and application of digital technologies in the agricultural and rural sectors has begun to bear fruit. Precision agriculture, smart agriculture using blockchain, artificial intelligence, remote sensing, sensors and other digital technologies are becoming increasingly mature in their application in the production chain, helping to accelerate the transformation of agricultural production methods and promoting the improvement of the quality and efficiency of agricultural production [47, 48]. Third, the rapid development of rural e-commerce, represented by Taobao villages, has brought about the agglomeration of agricultural factors and the popularization of green technology, which has brought opportunities to reduce the amount of chemical fertilizers and alleviate the pollution of agricultural surface sources [41].

In terms of the formation mechanism, in mechanism 1 "two-way FDI→green technology innovation→agricultural digitization and green growth" and mechanism 2 "two-way FDI→ overall technology innovation→agricultural digitization and green growth", two-way FDI has a significant positive impact on green technology innovation and overall technology innovation, passing the 1% significance test. The possible reason is that, on the one hand, the two-way FDI stimulates the agricultural digital transformation has a knowledge spillover effect on agricultural operators, through the dissemination of green production technology, improves the production quality of green agricultural products, suppresses the production of agricultural carbon emissions [41], and at the same time, improves the comprehensive quality of agricultural operators, helps them cultivate ecological and environmental protection awareness [42], and then promotes the integration of agricultural digitalization and green growth. On the other hand, two-way FDI greatly facilitates green technology innovation, agricultural scale operation and optimization of agricultural cultivation structure, which promotes green growth, and in turn promotes the convergence of digital and green growth in agriculture.

## 6. Conclusions and future research orientations

### 6.1 Spatial autocorrelation analysis

**Fig 2:**
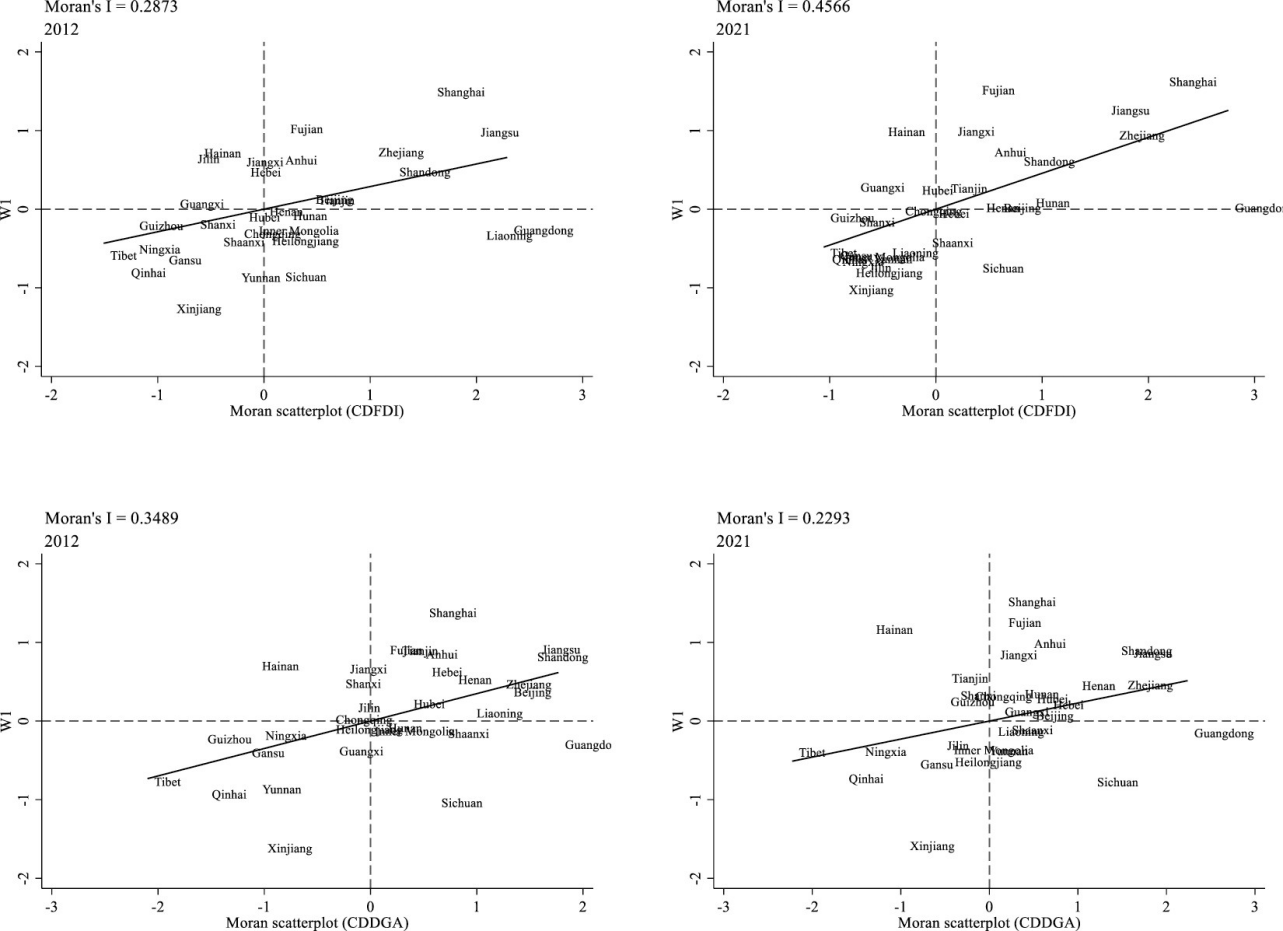
Moran scatter plot of two-way FDI development level (top) and the DGF level (bottom).

The existence of spatial correlation is an important prerequisite for spatial econometric analysis. Moran’s I index was used to measure the spatial correlation between the development level of two-way FDI and agricultural DGF under the 0–1 adjacent weight matrix, and the results are shown in Table 7. Among them, the global Moran’s I index of the development level of two-way FDI and agricultural DGF both passed the 5% significance test and were positive, indicating that the development level of two-way FDI and agricultural DGF in all 31 provinces from 2012 to 2021 showed positive spatial correlation. 2021, both the two-way FDI development level and agricultural DGF in 31 provinces show positive spatial correlation. This indicates that both tend to cluster in space and there is a "Matthew effect". It can be seen that each target variable has a significant positive spatial correlation, which is suitable for constructing a spatial measurement model. The Moran scatter plots of two-way FDI development level and agricultural DGF in some years are shown in Fig 2.

**Table 7:** Global Moran’s I index for the DGF and two-way FDI development level.

Year	DGF		CDFDI	
	Moran’s I	P-value	Moran’s I	P-value
2012	0.3489	0.0011	0.2873	0.0055
2013	0.3203	0.0024	0.3738	0.0005
2014	0.3188	0.0026	0.3118	0.0029
2015	0.3096	0.0032	0.4722	0.0000
2016	0.2761	0.0077	0.4586	0.0000
2017	0.2502	0.0145	0.4833	0.0000
2018	0.2127	0.0333	0.4593	0.0000
2019	0.2077	0.0371	0.4772	0.0000
2020	0.2322	0.0213	0.4541	0.0000
2021	0.2293	0.0231	0.4566	0.0000

### 6.2 Impact effect analysis

According to the test idea of Han Feng (2020) [49], this paper adopts the methods of "from specific to general" and "general to specific" to test the spatial econometric model. In addition, this paper draws on the spatiotemporal two-fixed-effects bias-corrected spatial Durbin model estimates proposed by Lee and Yu [50]. The results of the tests are shown in S4 File.

The results presented in Table 8 show the regression analysis of the two-way FDI on the agricultural DGF using different spatial weights. In columns (2) and (3), the spatial autoregressive coefficients of the spatial Durbin model (SDM) and the spatio-temporal Durbin model (STDM) are reported under the 0–1 adjacent weight matrix. Both coefficients, 0.607 for the SDM and 0.32 for the STDM, are statistically significant at the 1% level. This indicates that there is a positive spatial spillover effect on the agricultural DGF among the regions in China, which aligns with the results of the spatial correlation test mentioned earlier. Specifically, in the STDM, the direct effect of the two-way FDI on the agricultural DGF is significantly positive with a coefficient of 0.140, also significant at the 1% level. However, the spatial effect of the two-way FDI development level is not statistically significant with a coefficient of 0.107. In columns (5) and (6) of Table 8, the analysis is conducted using the economic geography matrix as the spatial weights. The spatial autoregressive coefficients for the SDM and the STDM are 0.658 and 0.325, respectively, and both are significant at the 1% level. This suggests a positive spatial spillover effect on agricultural DGF In the STDM, the direct effect of the two-way FDI development level on the agricultural DGF is significantly positive with a coefficient of 0.128, also significant at the 1% level. Overall, these results indicate the presence of a positive spatial spillover effect on the agricultural DGF among Chinese regions. The two-way FDI development level has a direct positive impact on the agricultural DGF, particularly in the spatio-temporal context. However, the spatial effect of the two-way FDI development level alone is not statistically significant, suggesting that other factors may also play a role in the spatial spillover effect.

**Table 8:** Global Moran’s I index for the DGF and two-way FDI development level.

Variables	W1			W2		
	(1)	(2)	(3)	(4)	(5)	(6)
W*DGF	-0.028	0.607***	0.320***	-0.042	0.658***	0.325***
	(-0.36)	(13.53)	(3.32)	(-0.22)	(8.33)	(2.91)
	0.466***	0.117**	0.140***	0.505***	0.143***	0.128***
	(4.83)	(2.09)	(2.73)	(5.91)	(3.58)	(2.93)
W*CDFDI	-0.139	-0.011	0.107	-0.483	-0.306**	-0.062
	(-0.98)	(-0.13)	(1.28)	(-1.36)	(-2.53)	(-0.31)
Sigma	0.001***	0.000***	0.000***	0.001***	0.000***	0.000***
	(5.96)	(8.34)	(8.82)	(6.13)	(8.28)	(7.59)
Observations	310	310	310	310	310	310
R^2^	0.806	0.062	0.009	0.688	0.023	0.057
Control Var	Yes	Yes	Yes	Yes	Yes	Yes
W*Control Var	Yes	Yes	Yes	Yes	Yes	Yes
Province FE	no	Yes	Yes	no	Yes	Yes
Year FE	Yes	no	Yes	Yes	no	Yes

* Values of z-statistics in parentheses are at heteroskedasticity robust standard errors.

*, **, and a at the 10%, 5%, and 1% levels, respectively. Same for the following tables.

### 6.3 Spatial effect decomposition

To further respond to the spatial spillover effects of the drivers and to avoid possible misinterpretation of the spatial spillover effects by simple point estimation results, the effects of each driver on the agricultural DGF were decomposed into direct and indirect effects according to the theory of Lesage & Pace (2008) [51]. The decomposition effects of each driver are shown in Table 9. The results show that (1) under the 0–1 adjacent weight matrix, the coefficient of indirect effect of two-way FDI development level is 0.208 and significantly positive at the 1% level, indicating that the growth of local two-way FDI development level improves the agricultural DGF in the surrounding areas to a certain extent. From a regional perspective, the effect of two-way FDI development level on the coordinated development level of agricultural digitalization and greening is significantly positive, which may be due to the fact that a higher coordinated development level of two-way FDI can provide financial support for the coordinated development level of agricultural digitalization and greening to a certain extent and effectively promote the R&D and transformation of green technological innovation results, thus promoting the coordinated development level of agricultural digitalization and greening. The development of the level of coordinated development of agricultural digitalization and greening. (2) Under the economic geography weight matrix, the increase of two-way FDI development level has a significant positive effect on the agricultural DGF in the region, while it has a weak negative effect on the agricultural DGF in regions with close per capita GDP development level, and its total effect on the agricultural DGF is also positive in a comprehensive view.

**Table 9:** Direct and indirect effects of the SDM model.

	W1			W2		
Variables	(1)	(2)	(3)	(4)	(5)	(6)
	Direct Effects	Indirect Effects	Total Effects	Direct Effects	Indirect Effects	Total Effects
CDFDI	0.154***	0.208*	0.362***	0.130***	-0.002	0.128
	(3.04)	(1.91)	(3.09)	(2.92)	(-0.01)	(0.40)
Observations	310	310	310	310	310	310
R^2^	0.009	0.009	0.009	0.057	0.057	0.057
Control Var	Yes	Yes	Yes	Yes	Yes	Yes
Province FE	Yes	Yes	Yes	Yes	Yes	Yes
Year FE	Yes	Yes	Yes	Yes	Yes	Yes

### 6.4 Conclusion and policy implications

To achieve the "double carbon" goal, digitalization is becoming a catalyst for transformation and upgrading, promoting the synergistic integration of the green economy and the digital economy. It is crucial to establish a sound policy that guides the import and export of FDI in both directions, enabling the agricultural DGF while enhancing economic efficiency. This paper presents the following conclusions based on empirical analysis: (1) Two-way FDI significantly contributes to agricultural DGF controlling for other variables, a 1% increase in the level of two-way FDI development, on average, results in a 0.1604 unit increase in the agricultural DGF and a 0.435 unit increase in the level of agricultural digitization. (2) Mechanism testing confirms that two-way FDI promotes the agricultural DGF by fostering green technology innovation capability and overall regional technology innovation capability. (3) Heterogeneity analysis reveals that two-way FDI significantly enhances the agricultural DGF in the eastern and central regions, while having a negative and insignificant effect on the western region. Additionally, compared to inland regions and regions with low economic development levels, two-way FDI has a relatively stronger promotion effect on the development level of agricultural digital greening in the same coastal regions and provinces with high economic development levels. (4) A positive spatial spillover effect was observed in the spatial Durbin model for the agricultural DGF among Chinese regions, indicating that the growth of local two-way FDI contributes to some extent to the agricultural DGF of neighboring regions. Furthermore, this paper conducted robustness tests and endogeneity tests by replacing the robust standard error clustering level, substituting core explanatory variables, and finding instrumental variables for 2SLS regression tests. The test results consistently demonstrate that the coordinated development level of two-way FDI significantly contributes to agricultural DGF.

Based on the aforementioned research, this paper offers the following policy recommendations: First, steadfastly follow the path of openness, enhance the top-level mechanism design for the coordinated development of digitalization and greening, provide reasonable guidance for foreign capital inflows, and actively explore replicable experiences in high-end industrial incubation. Strengthen the green and low-carbon focus of digital infrastructure, and utilize technologies such as 5G and artificial intelligence to systematically plan energy-saving and emission-reduction strategies for the entire life cycle of digital infrastructure. Second, fully leverage the role of technological innovation in enhancing agricultural DGF Encourage collaboration between industries, universities, and research institutes, support enterprises and research institutions in their digitalization and greening research and development efforts in agriculture, reform institutional mechanisms that impede scientific and technological innovation, improve support systems, and optimize the environment for scientific and technological innovation. Third, harness the spatial radiation function for the coordinated development of digitalization and greening. Establish digital infrastructure networks, ensure the smooth flow of data resources, and achieve seamless integration of software and hardware for the advancement of the digital economy. Concerning hardware facilities, accelerate the overall upgrading of digital economy infrastructure in city clusters through the development of strategic plans for the integration and coordinated development of digitalization and greening. Aim to comprehensively build a modern infrastructure system that is intensive and efficient, cost-effective and practical, intelligent and environmentally friendly, as well as safe and reliable. Regarding the soft service environment, cities with relatively limited digital economy development should actively attract high-level digital technology talent and engage in digital industrial software cooperation with central cities to collaboratively establish integrated industrial Internet platforms, industrial big data service centers, and smart supply chains.

Despite the research progress made, this study still has some limitations. For one, due to data collection constraints, the model in this study only uses data from 2012 to 2021. To further investigate the impact of two-way FDI on the growth of the convergence of digitization and greening in agriculture, a panel dataset with a larger sample is required for an acceptable empirical analysis. Second, this study only measured the level of digitalization and greening integration in agriculture in terms of digital foundation, popularization, application, human resources, innovation development, production efficiency, ecological environment, resource conditions, and negative environmental externalities, which may lead to a certain deviation of the measurement results from the current situation of digital agriculture development and green agriculture development in China. Future research can further improve the measurement system of digital agriculture and green agriculture, and with the continuous enrichment of relevant data, produce more accurate statistical results.

## Supporting information

S1 File The construction table of the indicator system for measuring the degree of agricultural DGF. (DOCX)

S2 File Data file. (XLS)

S3 File Stata 17.0 software run instructions. (DOCX)

S4 File The results of each test of the selected space model. (DOCX)

## References

[1] Dara R, Fard SMH, Kaur J. Recommendations for ethical and responsible use of artificial intelligence in digital agriculture. Frontiers in Artificial Intelligence. 2022;5. doi: 10.3389/frai.2022.884192 WOS:000915763600001. 35968036 PMC9372537

[2] Bhat SA, Huang N-F. Big Data and AI Revolution in Precision Agriculture: Survey and Challenges. Ieee Access. 2021;9:110209–22. doi: 10.1109/access.2021.3102227 WOS:000683992600001.

[3] Kalyani Y, Collier R. A Systematic Survey on the Role of Cloud, Fog, and Edge Computing Combination in Smart Agriculture. Sensors. 2021;21(17). doi: 10.3390/s21175922 WOS:000694485600001. 34502813 PMC8434609

[4] Krithika LB. Survey on the Applications of Blockchain in Agriculture. Agriculture-Basel. 2022;12(9). doi: 10.3390/agriculture12091333 WOS:000857447100001.

[5] Erik B, Andrew M. Race Against the Machine: How the Digital Revolution is Accelerating Innovation, Driving Productivity, and Irreversibly Transforming Employment and the Economy: Digital Frontier Press; 2012.

[6] Yin S, Yu Y. An adoption-implementation framework of digital green knowledge to improve the performance of digital green innovation practices for industry 5.0. J Clean Prod. 2022;363. doi: 10.1016/j.jclepro.2022.132608 WOS:000822709400003.

[7] Takalo SK, Tooranloo HS, Parizi ZS. Green innovation: A systematic literature review. J Clean Prod. 2021;279. doi: 10.1016/j.jclepro.2020.122474 WOS:000595933500006.

[8] Nylen D, Holmstrom J. Digital innovation in context Exploring serendipitous and unbounded digital innovation at the church of Sweden. Information Technology & People. 2019;32(3):696–714. doi: 10.1108/itp-05-2017-0148 WOS:000479216300010.

[9] Hou R, Li S, Chen H, Ren G, Gao W, Liu L. Coupling mechanism and development prospect of innovative ecosystem of clean energy in smart agriculture based on blockchain. J Clean Prod. 2021;319. doi: 10.1016/j.jclepro.2021.128466 WOS:000729130900008.

[10] Wang X, Liu Y, Bi J, Liu M. New challenges of the Belt and Road Initiative under China?s "3060" carbon target. J Clean Prod. 2022;376. doi: 10.1016/j.jclepro.2022.134180 WOS:000876691700007.

[11] Yang H, Li L, Liu Y. The effect of manufacturing intelligence on green innovation performance in China. Technological Forecasting and Social Change. 2022;178. doi: 10.1016/j.techfore.2022.121569 WOS:000778411300013.

[12] Bharadwaj A, El Sawy OA, Pavlou PA, Venkatraman N. Digital business strategy: toward a next generation of insights. Mis Quarterly. 2013;37(2):471–82. doi: 10.25300/misq/2013/37:2.3. WOS:000329754600009.

[13] Yoo Y, Boland RJ Jr, Lyytinen K, Majchrzak A. Organizing for Innovation in the Digitized World. Organization Science. 2012;23(5):1398–408. doi: 10.1287/orsc.1120.0771 WOS:000309096600012.

[14] Iansiti ML, Karim R. Digital Ubiquity: How Connections, Sensors, and Data Are Revolutionizing Business. Harvard Business Review. 2014;40(11):72–88. doi: 10.2469/dig.v45.n2.8

[15] Jacobides MGC, Carmelo Gawer, Annabelle. Towards a Theory of Ecosystems. Social Science Electronic Publishing. 2018;39. doi: 10.1002/smj.2904

[16] Porter M E HJE. How Smart, Connected Products Are Transforming Companies. Harvard Business Review. 2014;92:24–.

[17] Onitsuka K, Hoshino S. Inter-community networks of rural leaders and key people: Case study on a rural revitalization program in Kyoto Prefecture, Japan. J Rural Stud. 2018;61:123–36. doi: 10.1016/j.jrurstud.2018.04.008 WOS:000440120000010.

[18] Zhang HW H. R.; Li Z. Research on High Quality Development Evaluation of Digital Agriculture Under the Background of Rural Revitalization—Based on the Data Analysis of 31 Provinces and Cities in China From 2015 to 2019. Journal of Shaanxi Normal University (Philosophy and Social Sciences Edition). 2021;50(03):141–54. doi: 10.15983/j.cnki.sxss.2021.0525

[19] Sadjadi EN, Fernandez R. Challenges and Opportunities of Agriculture Digitalization in Spain. Agronomy-Basel. 2023;13(1). doi: 10.3390/agronomy13010259 WOS:000916776500001.

[20] Gargallo-Castel A, Esteban-Salvador L, Pérez-Sanz J. Impact of Gender in Adopting and Using ICTs in Spain. Journal of technology management & innovation. 2010;5(3):120–8. doi: 10.4067/s0718-27242010000300009 SCIELO:S0718-27242010000300009.

[21] Reinhardt T. The farm to fork strategy and the digital transformation of the agrifood sector-An assessment from the perspective of innovation systems. Applied Economic Perspectives and Policy. 2023;45(2):819–38. doi: 10.1002/aepp.13246 WOS:000766950900001.

[22] Bolfe EL, Jorge LAdC, Sanches IDA, Luchiari Junior A, da Costa CC, Victoria DdC, et al. Precision and Digital Agriculture: Adoption of Technologies and Perception of Brazilian Farmers. Agriculture-Basel. 2020;10(12). doi: 10.3390/agriculture10120653 WOS:000601731500001.

[23] Xia XL, Chen Z, Zhang Hl, Zhao MJ. Agricultural High-quality Development: Digital Empowerment and Implementation Path. Chinese Rural Economy. 2019;420(12):2–15.

[24] Cui H, Zhao T, Tao P. Evolutionary Game Study on the Development of Green Agriculture in China Based on Ambidexterity Theory Perspective. Polish Journal of Environmental Studies. 2019;28(3):1093–104. doi: 10.15244/pjoes/87139 WOS:000461800200007.

[25] Zhao HJ, Yu FW. Evaluation of Agricultural Green Development Level in Main Grain Producing Areas based on Entropy Method. Reform. 2019;11:136–46.

[26] Yu FW. An Analysis of the Reasons, Core and Countermeasures of Agricultural Green Development in the New Era. Chinese Rural Economy. 2018;05:19–34.

[27] Sun XYL Y.,. Can Land Trusteeship Improve Farmers’ Green Production? Chinese Rural Economy. 2019;10.

[28] Li MY, Chen K. An Empirical Analysis of Farmers’ Willingness and Behaviors in Green Agriculture Production. Journal of Huazhong Agricultural University(Social Sciences Edition). 2020;148(04):10–9+173–4. doi: 10.13300/j.cnki.hnwkxb.2020.04.002

[29] Li QJ, Liao CZ, Liu JH, Gao F, Lei QL. DEVELOPMENT OF QUALITY & SAFETY AGRICULTURAL PRODUCTS FROM THE PERSPECTIVE OF GREEN PRODUCTION. Chinese Journal of Agricultural Resources and Regional Planning. 2014;35(05):135–8.

[30] Dong YM Y. Y. The Path Selection and Efficiency Increase Mechanism of Farmers’ Adoption of Environmentally Friendly Technology: An Empirical Analysis. China Rural Survey. 2019;146(02):34–48.

[31] Genius M, Koundouri P, Nauges C, Tzouvelekas V. Information Transmission in Irrigation Technology Adoption and Diffusion: Social Learning, Extension Services, and Spatial Effects. Am J Agr Econ. 2014;96(1):328–44. doi: 10.1093/ajae/aat054 WOS:000330833100017.

[32] Li CX, Xu JB. Theoretical Interpretation and Practical Path of China’s Agricultural Green Transformation. Academic Journal of Zhongzhou. 2022;09:40–8.

[33] Santarius T, Dencik L, Diez T, Ferreboeuf H, Jankowski P, Hankey S, et al. Digitalization and Sustainability: A Call for a Digital Green Deal. Environmental Science & Policy. 2023;147:11–4. doi: 10.1016/j.envsci.2023.04.020 WOS:001011643600001.

[34] Gong MQ, Liu HY. The Influence of Two-way FDI Coordinated Development and Industrial Structure Evolution on Environmental Pollution in China. Journal of International Trade. 2020;02:110–24. doi: 10.13510/j.cnki.jit.2020.02.008

[35] Ahmed YAI, Roshna Ramzi;. The Impact of FDI Inflows and Outflows on Economic Growth: An Empirical Study of some Developed and Developing Countries. Journal of Raparin University. 2019;1. doi: 10.26750/VOL(6).NO(1).PAPER9

[36] Zhou GF, Dong ZY, Shen B. Human Capital Agglomeration, Digital Economy Development and "Industry-Consumption" Synergistic Upgrading. 45. 2023;06:70–84. doi: 10.13781/j.cnki.1007-9556.2023.06.006

[37] Wang H, Fang L. Analysis of the Spatio-temporal Coupling and Coordination Relationship and the Driving Forces of the Digitalization and Greening of Chinese Agriculture. Resources and Environment in the Yangtze Basin. 2023;32(04):868–82.

[38] Zou ZMC X. Research on the Coordinative Development Level and Influencing Factors of China’s Inter-provincial Two-way FDI Based on the Measurement of PVAR Model and Empirical Analysis of Dynamic Panel. Inquiry into Economic Issues. 2021;08:179–90.

[39] Yang L, Yang SH, Plotnick L. How the Internet of things technology enhances emergency response operations. Technological Forecasting and Social Change. 2013;80(9):1854–67. doi: 10.1016/j.techfore.2012.07.011 WOS:000326427400015.

[40] Jiang T. Mediating Effects and Moderating Effects in Causal Inference. China Industrial Economics. 2022;410(05):100–20. doi: 10.19581/j.cnki.ciejournal.2022.05.005

[41] Wang HR, Cui HR, Zhao QZ. Effect of green technology innovation on green total factor productivity in China: Evidence from spatial durbin model analysis. J Clean Prod. 2021;288. doi: 10.1016/j.jclepro.2020.125624 WOS:000631538800062.

[42] Adnan N, Nordin SM, Ali M. A solution for the sunset industry: Adoption of Green Fertiliser Technology amongst Malaysian paddy farmers. Land Use Policy. 2018;79:575–84. doi: 10.1016/j.landusepol.2018.08.033 WOS:000454378800051.

[43] Jin Y, Wen W, He Y. Impact of Digital Transformation on Corporate Green Innovation:Evidence from China’s Manufacturing Listed Companies. Finance and Trade Research. 2022;33(07). doi: 10.19337/j.cnki.34-1093/f.2022.07.006

[44] Cao Y, Li X, Hu HL, Wan GY, Wang SY. How Does Digitalization Drive the Green Transformation in Manufacturing Companies? An Exploratory Case Study from the Perspective of Resource Orchestration Theory. Journal of Management World. 2023;39(03):96–112+26+3. doi: 10.19744/j.cnki.11-1235/f.2023.0045

[45] Jiang Q, Li JZ, Si HY, Su YY. The Impact of the Digital Economy on Agricultural Green Development: Evidence from China. Agriculture-Basel. 2022;12(8). doi: 10.3390/agriculture12081107 WOS:000846424200001.

[46] Shen ZY, Wang SK, Boussemart JP, Hao Y. Digital transition and green growth in Chinese agriculture. Technological Forecasting and Social Change. 2022;181. doi: 10.1016/j.techfore.2022.121742 WOS:000822688900019.

[47] Wu F. Adoption and income effects of new agricultural technology on family farms in China. PLoS One. 2022;17(4). doi: 10.1371/journal.pone.0267101 WOS:000816171900026. 35472213 PMC9041835

[48] Yang CH. Remote Sensing and Precision Agriculture Technologies for Crop Disease Detection and Management with a Practical Application Example. Engineering. 2020;6(5):528–32. doi: 10.1016/j.eng.2019.10.015 WOS:000541483200012.

[49] Han FY L. G.,. How Does the Agglomeration of Producer Services Promote the Upgrading of Manufacturing Structure?:An Integrated Framework of Agglomeration Economies and Schumpeter’s Endogenous Growth Theory. Journal of Management World. 2020;36(02):72–94. doi: 10.19744/j.cnki.11-1235/f.2020.0022

[50] Lee L-f Yu J. Estimation of spatial autoregressive panel data models with fixed effects. J Econom. 2010;154(2):165–85. doi: 10.1016/j.jeconom.2009.08.001 WOS:000273622900006.

[51] LJP. An Introduction to Spatial Econometrics. Revue d économie industrielle. 2008;123:513–4. doi: 10.4000/rei.3887

