# Supplementary material for: Two-Way FDI assists agricultural sustainable development: Based on digitalization and greening perspectives
Source: PLoS One. 2024 Feb 16;19(2):e0296896. doi: 10.1371/journal.pone.0296896 (PMC10871478; doi:10.1371/journal.pone.0296896)
Supplement: S1 File — (DOCX) [file pone.0296896.s001.docx]

Table 10 shows the construction table of the indicator system for measuring the degree of agricultural DGF.

**Table 10.** Digitalization and Greening of Chinese Agriculture Indicator System.

| Target Level | Guideline level | Indicator Level | Calculating formula |
| --- | --- | --- | --- |
| Degree of digital development in agriculture | Digital Foundation | Postal Network Points (Service) | |
|  |  | Telephone penetration rate (%) | |
|  |  | Length of fiber optic cable (km) | |
|  | Digital Popularity | Number of 3G cell phones (million) | |
|  |  | Number of Internet broadband access ports (million) | |
|  |  | Number of broadband access subscribers (million) | |
|  | Digital Applications | Total post and telecommunications business (billion yuan, comparable prices) | |
|  |  | Express delivery business volume (million pieces) | |
|  |  | Cargo volume (million tons) | |
|  | Talent Resources | Enrollment in higher education institutions (persons) | |
|  |  | Average number of students enrolled in higher education per 100,000 population (persons) | |
|  |  | Number of research R&D personnel (persons) | |
|  | Innovative Development | Number of patents granted (pieces) | |
|  |  | Total turnover of technology contracts (million yuan) | |
|  |  | Number of patent applications received (pieces) | |
|  |  | Internal expenditure on R&D expenses for research and experimental development (million yuan) | |
| The degree of green development of agriculture | Production efficiency | Level of agricultural development | Primary GDP/GDP |
|  |  | Total agricultural output | Primary GDP |
|  |  | Grain unit area yield | Grain yield per unit area |
|  |  | Total factor productivity in agriculture | Calculated from the table below |
|  | Ecosystem | Soil erosion control efforts | Soil erosion control area |
|  |  | Forest coverage | Forest coverage |
|  |  | Agricultural disaster rate | Agricultural disaster area/ cultivated land |
|  | Resource conditions | Total mechanical power per unit of cultivated land area | Total power of farm machinery / cultivated land |
|  |  | Water-saving irrigation rate | Water-saving irrigation area / cultivated land |
|  |  | Agricultural water | Agricultural water use |
|  | Negative environmental externalities | Total Nitrogen Emission Intensity | Total nitrogen pollution emissions / cultivated land |
|  |  | Total phosphorus pollution emission intensity | Total phosphorus pollution emissions / cultivated land |
|  |  | Pesticide application strength | Pesticide application rate/cultivated area |
|  |  | Film usage strength | Amount of agricultural film used/cultivated land |
|  |  | Agricultural carbon intensity | Agricultural carbon emissions/cultivated land |

Total factor productivity of agriculture can effectively measure the efficiency of agricultural production and can be used as an important representation of the transformation of agricultural growth momentum and innovation drive. Referring to the research of scholars, this paper takes agriculture in a narrow sense, i.e., plantation industry, and agricultural labor, land, fertilizer, agricultural machinery, and irrigation as input indicators, among which the total agricultural output value is weighted as the proportion of the total agricultural, forestry, animal husbandry and fishery output value, and the total agricultural output value is used as output indicator. In this study, the GML index based on the SBM model was used to calculate the total factor productivity of Chinese agriculture using MaxDEA Ultra 8 software.

**Table 11.** Total factor productivity measurement system in agriculture.

| Primary Indicator | Secondary Indicator | Calculation Formula |
| --- | --- | --- |
| Input Indicators | Agricultural labor input | Agricultural workers (10,000 people) |
|  | Land input | Crop sown area (thousand/hm2) |
|  | Fertilizer input | Fertilizer application in agriculture folded pure (million tons) |
|  | Pesticide input | Pesticide use (million tons) |
|  | Agricultural machinery inputs | Total power of agricultural machinery (million/kw.h) |
|  | Agricultural Water Resources Inputs | Effective irrigated area (thousand/hm2 ) |
| Output Indicators | Total agricultural output | Total agricultural output value ( constant 2000 prices, billion yuan) |
