# Supplementary material for: Two-Way FDI assists agricultural sustainable development: Based on digitalization and greening perspectives
Source: PLoS One. 2024 Feb 16;19(2):e0296896. doi: 10.1371/journal.pone.0296896 (PMC10871478; doi:10.1371/journal.pone.0296896)
Supplement: S3 File — (DOCX) [file pone.0296896.s003.docx]

import excel "05. new data.xls", sheet("Sheet1") firstrow

**# 描述性统计分析

sum y1 y2 y3 x1 x2 c3 c5 c6 c7 c9 m1 m2 m3 m5

logout, save("描述性统计") word replace: ///

tabstat y1 y2 y3 x1 x2 c3 c5 c6 c7 c9 m1 m2 m3 m5 , stats(n mean sd min p50 max) format(%10.4f) column(stats)

global cv "c3 c5 c6 c7 c9"

**# 基准回归

reghdfe y1 x1 ,absorb(pid year) vce(robust) //系数：0.14；P值：0.001

outreg2 using 基准回归.doc,replace tstat bdec(4) tdec(4) rdec(4) ctitle(y1) addtext(province fe, yes,year fe, yes)

reghdfe y2 x1 ,absorb(pid year) vce(robust) //系数：0.579；P值：0.000

outreg2 using 基准回归.doc,append tstat bdec(4) tdec(4) rdec(4) ctitle(y2) addtext(province fe, yes,year fe, yes)

reghdfe y3 x1 ,absorb(pid year) vce(robust) //不显著

outreg2 using 基准回归.doc,append tstat bdec(4) tdec(4) rdec(4) ctitle(y3) addtext(province fe, yes,year fe, yes)

reghdfe y1 x1 $cv ,absorb(pid year) vce(robust) //系数：0.1；P值：0.011

outreg2 using 基准回归.doc,append tstat bdec(4) tdec(4) rdec(4) ctitle(y1) addtext(province fe, yes,year fe, yes)

reghdfe y2 x1 $cv ,absorb(pid year) vce(robust) //系数：0.422；P值：0.000

outreg2 using 基准回归.doc,append tstat bdec(4) tdec(4) rdec(4) ctitle(y2) addtext(province fe, yes,year fe, yes)

reghdfe y3 x1 $cv ,absorb(pid year) vce(robust) //系数：0.004；P值：0.938。不显著

outreg2 using 基准回归.doc,append tstat bdec(4) tdec(4) rdec(4) ctitle(y3) addtext(province fe, yes,year fe, yes)

**# 机制分析

**# （1）产业结构合理化（抑制合理化，显著）

reghdfe m1 x1 $cv ,absorb(pid year) vce(robust) //系数：-0.031；P值：0.076

outreg2 using 机制分析.doc,replace tstat bdec(4) tdec(4) rdec(4) ctitle(m1) addtext(province fe, yes,year fe, yes)

**# （2）产业结构高级化（促进高级化，不显著）

reghdfe m2 x1 $cv ,absorb(pid year) vce(robust) //不显著

outreg2 using 机制分析.doc,append tstat bdec(4) tdec(4) rdec(4) ctitle(m2) addtext(province fe, yes,year fe, yes)

**# （3）绿色创新数量（促进绿色创新，显著）

reghdfe m3 x1 $cv ,absorb(pid year) vce(robust) //系数：2.13；P值：0.000

outreg2 using 机制分析.doc,append tstat bdec(4) tdec(4) rdec(4) ctitle(m3) addtext(province fe, yes,year fe, yes)

**# （4）创新数量（促进整体创新，显著）

reghdfe m5 x1 $cv ,absorb(pid year) vce(robust) //系数：2.113；P值：0.000

outreg2 using 机制分析.doc,append tstat bdec(4) tdec(4) rdec(4) ctitle(m5) addtext(province fe, yes,year fe, yes)

**# 稳健性分析

**# （1）省份聚类稳健标准误

reghdfe y1 x1 $cv ,absorb(pid year) vce(cluster pid)

outreg2 using 稳健性分析.doc,replace tstat bdec(4) tdec(4) rdec(4) ctitle(y1) addtext(province fe, yes,year fe, yes)

**# （2）替换X

reghdfe y1 x2 $cv , absorb(pid year) vce(robust) //系数：1.628；P值：0.027

outreg2 using 稳健性分析.doc,append tstat bdec(4) tdec(4) rdec(4) ctitle(y1) addtext(province fe, yes,year fe, yes)

**# 内生性分析（2SLS）利用双向FDI的一阶和二阶滞后

eststo clear

xtset pid year

tab year, gen(dyear)

xtivreg2 y1 (x1=L.x1 L2.x1) $cv dyear*,fe robust first endog(x1)

xtivreg2 y1 (x1=L.x1 L2.x1) $cv dyear*,fe first savefp(first) robust //保存第一阶段结果

eststo second //保存第二阶段结果

est restore firstx1 //激活第一阶段结果

outreg2 using 内生性检验.doc,replace cttop(first) tstat bdec(4) tdec(4) rdec(4) addtext(province fe, yes,year fe, yes)

est restore second //激活第二阶段结果

outreg2 using 内生性检验.doc, append cttop(second) tstat bdec(4) tdec(4) rdec(4) addtext(province fe, yes,year fe, yes)

/*

1,Underidentification test (Kleibergen-Paap rk LM statistic):14.454;Chi-sq(2) P-val =0.0007

2,

Weak identification test (Cragg-Donald Wald F statistic): 22.104

(Kleibergen-Paap rk Wald F statistic): 10.075

Stock-Yogo weak ID test critical values: 10% maximal IV size 19.93

15% maximal IV size 11.59

20% maximal IV size 8.75

25% maximal IV size 7.25

3,Hansen J statistic (overidentification test of all instruments):0.052;Chi-sq(1) P-val =0.8188

*/

**# 异质性分析

**# 1. 区域异质性

gen region=1 if provname=="北京市"|provname=="天津市"|provname=="河北省"|provname=="辽宁省"|provname=="上海市"|provname=="江苏省"|provname=="浙江省"|provname=="福建省"|provname=="山东省"|provname=="广东省"|provname=="海南省"

replace region=2 if provname=="山西省"|provname=="吉林省"|provname=="黑龙江省"|provname=="安徽省"|provname=="江西省"|provname=="河南省"|provname=="湖北省"|provname=="湖南省"

replace region=3 if provname=="四川省"|provname=="重庆市"|provname=="云南省"|provname=="西藏自治区"|provname=="陕西省"|provname=="甘肃省"|provname=="青海省"|provname=="宁夏回族自治州"|provname=="新疆维吾尔自治区"|provname=="广西壮族自治区"|provname=="内蒙古自治区"

reghdfe y1 x1 $cv if region==1 ,absorb(pid year) vce(robust) //系数：0.1；P值：0.011

outreg2 using 异质性.doc,replace tstat bdec(4) tdec(4) rdec(4) ctitle(y1) addtext(province fe, yes,year fe, yes)

reghdfe y1 x1 $cv if region==2 ,absorb(pid year) vce(robust) //系数：0.1；P值：0.011

outreg2 using 异质性.doc,append tstat bdec(4) tdec(4) rdec(4) ctitle(y1) addtext(province fe, yes,year fe, yes)

reghdfe y1 x1 $cv if region==3 ,absorb(pid year) vce(robust) //系数：0.1；P值：0.011

outreg2 using 异质性.doc,append tstat bdec(4) tdec(4) rdec(4) ctitle(y1) addtext(province fe, yes,year fe, yes)

**# 2. 沿海和内陆

inlist2 provname,values(辽宁省,河北省,天津市,山东省,江苏省,上海市,浙江省,福建省,广东省,广西壮族自治区,海南省) name(seadum)

replace seadum=0 if seadum==.

label variable seadum "沿海=1；内陆=0"

reghdfe y1 c.x1#i.seadum $cv ,absorb(pid year) vce(robust) //系数：0.1；P值：0.011

outreg2 using 异质性.doc,append tstat bdec(4) tdec(4) rdec(4) ctitle(y1) addtext(province fe, yes,year fe, yes)

**# 3. 经济发展水平（中位数）

egen mgdp=median(gddp)

sort pid year

bys pid: egen mcgdp=median(gddp)

gen gdpdum=1 if mcgdp>mgdp

replace gdpdum=0 if gdpdum==.

drop mgdp mcgdp

label variable gdpdum "经济发展水平高=1；经济发展水平低=0"

reghdfe y1 c.x1#i.gdpdum $cv ,absorb(pid year) vce(robust) //系数：0.1；P值：0.011

outreg2 using 异质性.doc,append tstat bdec(4) tdec(4) rdec(4) ctitle(y1) addtext(province fe, yes,year fe, yes)

**# 进一步检验--空间计量SDM

clear all

cd D:\Documents\Desktop\农业数绿耦合度与双向FDI\OLS

spatwmat using 邻接矩阵.dta ,n(W1) standardize

spatwmat using 经济地理矩阵.dta ,n(W2) standardize

****START**

***********空间计量分析***********

use "temp.dta" ,clear

xtset pid year

**(1)空间自相关检验

***（1.1）全局莫兰指数

asdoc xtmoran y1, wname(邻接矩阵.dta) //显著

/*

Moran's I (varname : y1) Number of obs = 310

Group variable: pid Number of groups = 31

Time variable: year Panel length = 10

--------------------------------------------------------------

year | I E(I) Sd(I) Z P-value

--------------------+-----------------------------------------

2012 | 0.3489 -0.0333 0.1170 3.2673 0.0011

2013 | 0.3203 -0.0333 0.1166 3.0331 0.0024

2014 | 0.3188 -0.0333 0.1168 3.0164 0.0026

2015 | 0.3096 -0.0333 0.1163 2.9485 0.0032

2016 | 0.2761 -0.0333 0.1161 2.6649 0.0077

2017 | 0.2502 -0.0333 0.1159 2.4453 0.0145

2018 | 0.2127 -0.0333 0.1156 2.1290 0.0333

2019 | 0.2077 -0.0333 0.1156 2.0847 0.0371

2020 | 0.2322 -0.0333 0.1154 2.3019 0.0213

2021 | 0.2293 -0.0333 0.1156 2.2719 0.0231

--------------------------------------------------------------

*/

asdoc xtmoran x1, wname(邻接矩阵.dta) //显著

/*

Moran's I (varname : x1) Number of obs = 310

Group variable: pid Number of groups = 31

Time variable: year Panel length = 10

--------------------------------------------------------------

year | I E(I) Sd(I) Z P-value

--------------------+-----------------------------------------

2012 | 0.2873 -0.0333 0.1156 2.7738 0.0055

2013 | 0.3738 -0.0333 0.1161 3.5055 0.0005

2014 | 0.3118 -0.0333 0.1158 2.9794 0.0029

2015 | 0.4722 -0.0333 0.1153 4.3835 0.0000

2016 | 0.4586 -0.0333 0.1166 4.2199 0.0000

2017 | 0.4833 -0.0333 0.1167 4.4263 0.0000

2018 | 0.4593 -0.0333 0.1164 4.2328 0.0000

2019 | 0.4772 -0.0333 0.1166 4.3781 0.0000

2020 | 0.4541 -0.0333 0.1155 4.2217 0.0000

2021 | 0.4566 -0.0333 0.1143 4.2859 0.0000

--------------------------------------------------------------

*/

**#（1.2）局部莫兰指数及其散点图

xtmoran y1, wname(邻接矩阵.dta) morani(2012 2021) symbol(provname) graph //显著

xtmoran x1, wname(邻接矩阵.dta) morani(2012 2021) symbol(provname) graph //显著

xtmoran y1, wname(邻接矩阵.dta) morani(2012 2021) symbol(pengname) graph //显著

xtmoran x1, wname(邻接矩阵.dta) morani(2012 2021) symbol(pengname) graph //显著

***********空间计量分析***********

**# （1）静态SDM模型

//////邻接矩阵的基准回归--W1

global cv "c3 c5 c6 c7 c9"

spatwmat using 邻接矩阵.dta ,n(W1) standardize

xsmle y1 x1 $cv ,fe model(sdm) wmat(W1) type(time) nolog noeffects robust

outreg2 using 空间杜宾-基准回归.doc,replace tstat bdec(3) tdec(2) ctitle(y1) addtext(province fe, no,year fe, yes)

xsmle y1 x1 $cv ,fe model(sdm) wmat(W1) type(ind) nolog noeffects robust

outreg2 using 空间杜宾-基准回归.doc,append tstat bdec(3) tdec(2) ctitle(y1) addtext(province fe, yes,year fe, no)

xsmle y1 x1 $cv ,fe model(sdm) wmat(W1) type(both) nolog noeffects robust

outreg2 using 空间杜宾-基准回归.doc,append tstat bdec(3) tdec(2) ctitle(y1) addtext(province fe, yes,year fe, yes)

//////经济地理矩阵的基准回归--W2

spatwmat using 经济地理矩阵.dta ,n(W2) standardize

xsmle y1 x1 $cv ,fe model(sdm) wmat(W2) type(time) nolog noeffects robust

outreg2 using 空间杜宾-基准回归.doc,append tstat bdec(3) tdec(2) ctitle(y1) addtext(province fe, no,year fe, yes)

xsmle y1 x1 $cv ,fe model(sdm) wmat(W2) type(ind) nolog noeffects robust

outreg2 using 空间杜宾-基准回归.doc,append tstat bdec(3) tdec(2) ctitle(y1) addtext(province fe, yes,year fe, no)

xsmle y1 x1 $cv ,fe model(sdm) wmat(W2) type(both) nolog noeffects robust

outreg2 using 空间杜宾-基准回归.doc,append tstat bdec(3) tdec(2) ctitle(y1) addtext(province fe, yes,year fe, yes)

**# （2）空间分解

//////邻接矩阵的基准回归--W1

spatwmat using 邻接矩阵.dta ,n(W1) standardize

xsmle y1 x1 $cv ,fe model(sdm) wmat(W1) type(both) nolog effects robust

outreg2 using 空间杜宾-空间分解.doc,replace tstat bdec(3) tdec(2) ctitle(y1) addtext(province fe, yes,year fe, yes)

//////经济地理矩阵的基准回归--W2

spatwmat using 经济地理矩阵.dta ,n(W2) standardize

xsmle y1 x1 $cv ,fe model(sdm) wmat(W2) type(both) nolog effects robust

outreg2 using 空间杜宾-空间分解.doc,append tstat bdec(3) tdec(2) ctitle(y1) addtext(province fe, yes,year fe, yes)

************************************

************************************

************************************

************************************

************************************

************************************

************************************

************************************

************************************

**（2）模型选择

**（2.1）LM检验：判断是否存在空间效应 ,使用何种空间计量模型

//////邻接矩阵的LM检验--W1

use 邻接矩阵.dta ,clear

spmat dta W_1 x1-x31 ,replace

spcs2xt x1-x31 ,matrix(Wxt_01) time(10)

save Wxt_01.dta ,replace //保存LM检验所需空间面板权重矩阵

spatwmat using Wxt_01.dta ,name(Wxt_01m) standardize

use "temp.dta" ,clear

xtset pid year

reg y1 x1 $cv

spatdiag ,w(Wxt_01m) //LM检验

/*

Diagnostics

------------------------------------------------------------

Test | Statistic df p-value

-------------------------------+----------------------------

Spatial error: |

Moran's I | 7.705 1 0.000

Lagrange multiplier | 54.351 1 0.000

Robust Lagrange multiplier | 16.563 1 0.000

|

Spatial lag: |

Lagrange multiplier | 56.807 1 0.000

Robust Lagrange multiplier | 19.019 1 0.000

------------------------------------------------------------

*/

//////经济地理矩阵--W2

use 经济地理矩阵.dta ,clear

spmat dta W_2 r1-r31 ,replace

spcs2xt r1-r31 ,matrix(Wxt_02) time(10)

save "Wxt_02.dta" ,replace

spatwmat using Wxt_02.dta ,name(Wxt_02m) standardize

use "temp.dta" ,clear

xtset pid year

reg y1 x1 $cv

spatdiag ,w(Wxt_02m) //LM检验

/*

Diagnostics

------------------------------------------------------------

Test | Statistic df p-value

-------------------------------+----------------------------

Spatial error: |

Moran's I | 15.793 1 0.000

Lagrange multiplier | 214.194 1 0.000

Robust Lagrange multiplier | 92.097 1 0.000

|

Spatial lag: |

Lagrange multiplier | 147.418 1 0.000

Robust Lagrange multiplier | 25.321 1 0.000

------------------------------------------------------------

*/

**（2.2）HUASMAN检验：判断应该使用固定效应还是随机效应

//////邻接矩阵的HAUSMAN检验--W1

use "temp.dta" ,clear

xtset pid year

spatwmat using 邻接矩阵.dta ,n(W1) standardize

xsmle y1 x1 $cv ,model(sdm) wmat(W1) hausman nolog

/*

Ho: difference in coeffs not systematic chi2(13) = 21.46 Prob>=chi2 = 0.0644

*/

//////经济地理矩阵的HAUSMAN检验--W2

use "temp.dta" ,clear

xtset pid year

spatwmat using 经济地理矩阵.dta ,n(W2) standardize

xsmle y1 x1 $cv ,model(sdm) wmat(W2) hausman nolog

/*

Ho: difference in coeffs not systematic chi2(13) = 29.04 Prob>=chi2 = 0.0065

*/

**（2.3）LR检验：判断应该使用何种固定效应模型？

//////邻接矩阵的LR检验--W1

use "temp.dta" ,clear

xtset pid year

spatwmat using 邻接矩阵.dta ,n(W1) standardize

xsmle y1 x1 $cv ,fe model(sdm) wmat(W1) type(ind) nolog noeffects

est store sdm_ind1

xsmle y1 x1 $cv ,fe model(sdm) wmat(W1) type(time) nolog noeffects

est store sdm_time1

xsmle y1 x1 $cv ,fe model(sdm) wmat(W1) type(both) nolog noeffects

est store sdm_both1

lrtest sdm_both1 sdm_time1 ,df(16) //比较both与time模型

/*

Likelihood-ratio test

Assumption: sdm_time1 nested within sdm_both1

LR chi2(16) = 518.39

Prob > chi2 = 0.0000

*/

lrtest sdm_both1 sdm_ind1 ,df(16) //比较both与ind模型

/*

Likelihood-ratio test

Assumption: sdm_ind1 nested within sdm_both1

LR chi2(16) = 32.34

Prob > chi2 = 0.0090

*/

//////经济地理矩阵的LR检验--W2

use "temp.dta" ,clear

xtset pid year

spatwmat using 经济地理矩阵.dta ,n(W2) standardize

xsmle y1 x1 $cv ,fe model(sdm) wmat(W2) type(ind) nolog noeffects

est store sdm_ind2

xsmle y1 x1 $cv ,fe model(sdm) wmat(W2) type(time) nolog noeffects

est store sdm_time2

xsmle y1 x1 $cv ,fe model(sdm) wmat(W2) type(both) nolog noeffects

est store sdm_both2

lrtest sdm_both2 sdm_time2 ,df(16) //比较both与time模型

/*

Likelihood-ratio test

Assumption: sdm_time2 nested within sdm_both2

LR chi2(16) = 528.33

Prob > chi2 = 0.0000

*/

lrtest sdm_both2 sdm_ind2 ,df(16) //比较both与ind模型

/*

Likelihood-ratio test

Assumption: sdm_ind2 nested within sdm_both2

LR chi2(16) = 30.22

Prob > chi2 = 0.0169

*/

**（2.4）LR检验：判断空间杜宾模型的双固定效应是否优于空间误差和空间滞后？

//////邻接矩阵的LR检验--W1

spatwmat using 邻接矩阵.dta ,n(W1) standardize

xsmle y1 x1 $cv ,fe model(sar) wmat(W1) type(both) nolog noeffects

est store sar_both1

xsmle y1 x1 $cv ,fe model(sem) emat(W1) type(both) nolog noeffects

est store sem_both1

xsmle y1 x1 $cv ,fe model(sdm) wmat(W1) type(both) nolog noeffects

est store sdm_both1

lrtest sdm_both1 sar_both1 //比较sdm与sar模型

/*

Likelihood-ratio test

Assumption: sar_both1 nested within sdm_both1

LR chi2(6) = 49.50

Prob > chi2 = 0.0000

*/

lrtest sdm_both1 sem_both1 //比较sdm与sem模型

/*

Likelihood-ratio test

Assumption: sem_both1 nested within sdm_both1

LR chi2(6) = 45.03

Prob > chi2 = 0.0000

*/

//////经济地理矩阵的LR检验--W2

spatwmat using 经济地理矩阵.dta ,n(W2) standardize

xsmle y1 x1 $cv ,fe model(sar) wmat(W2) type(both) nolog noeffects

est store sar_both2

xsmle y1 x1 $cv ,fe model(sem) emat(W2) type(both) nolog noeffects

est store sem_both2

xsmle y1 x1 $cv ,fe model(sdm) wmat(W2) type(both) nolog noeffects

est store sdm_both2

lrtest sdm_both2 sar_both2 //比较sdm与sar模型

/*

Likelihood-ratio test

Assumption: sar_both2 nested within sdm_both2

LR chi2(6) = 48.80

Prob > chi2 = 0.0000

*/

lrtest sdm_both2 sem_both2 //比较sdm与sem模型

/*

Likelihood-ratio test

Assumption: sem_both2 nested within sdm_both2

LR chi2(6) = 50.72

Prob > chi2 = 0.0000

*/

**（2.5）Wald检验：判断空间杜宾模型是否会退化为空间误差和空间滞后？

//////邻接矩阵的Wald检验--W1

spatwmat using 邻接矩阵.dta ,n(W1) standardize

xsmle y1 x1 $cv ,fe model(sdm) wmat(W1) nolog effects robust

test [Wx]x1 =[Wx]c3 =[Wx]c5 =[Wx]c6 =[Wx]c7 =[Wx]c9

testnl ([Wx]x1=-[Spatial]rho*[Main]x1) ([Wx]c3=-[Spatial]rho*[Main]c3)([Wx]c5=-[Spatial]rho*[Main]c5)([Wx]c6=-[Spatial]rho*[Main]c6)([Wx]c7=-[Spatial]rho*[Main]c7)([Wx]c9=-[Spatial]rho*[Main]c9) //Wald检验

/*

chi2( 5) = 50.02

Prob > chi2 = 0.0000

*/

//////经济地理矩阵的Wald检验--W2

spatwmat using 经济地理矩阵.dta ,n(W2) standardize

xsmle y1 x1 $cv ,fe model(sdm) wmat(W2) nolog effects robust

test [Wx]x1 =[Wx]c3 =[Wx]c5 =[Wx]c6 =[Wx]c7 =[Wx]c9

testnl ([Wx]x1=-[Spatial]rho*[Main]x1) ([Wx]c3=-[Spatial]rho*[Main]c3)([Wx]c5=-[Spatial]rho*[Main]c5)([Wx]c6=-[Spatial]rho*[Main]c6)([Wx]c7=-[Spatial]rho*[Main]c7)([Wx]c9=-[Spatial]rho*[Main]c9) //Wald检验

/*

chi2(7) = 27.17

Prob > chi2 = 0.0001

*/
