# Supplementary material for: Two-Way FDI assists agricultural sustainable development: Based on digitalization and greening perspectives
Source: PLoS One. 2024 Feb 16;19(2):e0296896. doi: 10.1371/journal.pone.0296896 (PMC10871478; doi:10.1371/journal.pone.0296896)
Supplement: S4 File — (DOCX) [file pone.0296896.s004.docx]

From the test results, the LM-lag, LM-error, Robust LM-lag and Robust LM-error statistics in the LM test are all significant at 1% confidence level, indicating that the constructed model should include both spatial lag effect and spatial error effect, and therefore the more general spatial Durbin model should be used. The LR test and Wald test were further performed, and both statistics rejected the original hypothesis at the 1% confidence level, indicating that the spatial Durbin model cannot be degraded to a spatial lag model or a spatial error model, so the spatial Durbin model is more suitable for the study in this paper. In addition, both the temporal LR test and the spatial LR test reject the original hypothesis at the 1% confidence level, and the Hausman test also rejects the original hypothesis, indicating that the spatial Durbin model should be used for regression estimation in the paper(Table 9).

**Table 12.** The results of each test of the selected space model.

| Name of test | W1 | W2 |
| --- | --- | --- |
| LM- error | 54.351***  (0.000) | 214.194***  (0.000) |
| Robust LM- error | 16.563***  (0.000) | 92.097***  (0.000) |
| LM- lag | 56.807***  (0.000) | 147.418***  (0.000) |
| Robust LM- lag | 19.019***  (0.000) | 25.321***  (0.000) |
| LR_Spacial_ error | 49.50***  (0.000) | 48.80***  (0.000) |
| LR_Spacial_lag | 45.03***  (0.000) | 50.72***  (0.000) |
| Wald Test | 50.02***  (0.000) | 27.17***  (0.0001) |
| LR test (time) | 518.39***  (0.000) | 528.33***  (0.000) |
| LR test (space) | 32.34***  (0.0090) | 30.22**  (0.0169) |
| Hausman Test | 21.46*  (0.0644) | 29.04***  (0.0065) |
